# The Middlesex Electrical Installation

**Published:** 1903-05-30

**Authors:** 


### THE MIDDLESEX ELECTRICAL INSTALLATION.

Special hospitals are nowadays common enough, if indeed not much too common, but a couple of centuries ago only three classes of hospitals were recognised—general, lying-in, and lock hospitals. It was practically as a lying-in hospital that the Middlesex Hospital started its career, but only a year or two later a prescient philanthropist made a large donation to its funds upon certain conditions. These were that a ward should be opened and maintained in which women afflicted with cancer could be treated and, if necessary, remain until their death. The offer was accepted, and ever since then cancer treatment has been a specialty of an otherwise general hospital. With this history behind it the Middlesex Hospital has naturally been ever anxious to take advantage of every advance, real or hypothetical, that has been made in cancer treatment, and to some extent has experimented with electricity for a considerable time past. Towards the end of last year, however, it was determined to carry on such experiments in a more elaborate way, and the electrical installation now housed in a special building in the forecourt of the hospital is the result. This building is of galvanised iron outside, but fitted with wood inside in such fashion as to resemble any ordinary out-patient department. A cheap but neat building, in every way fitted for its purpose, it has a waiting-room, central consulting-room, and two annexes. It is in charge of Mr. Lyster, who attends daily at 11 A.M. with a qualified assistant; while there are two nurses attached to the department permanently, and other nurses who attend for instruction.

The chief source of electricity is a connection with the main, a pressure of 200 volts being connected with a switchboard where it can readily be subdivided as required. Besides this there are ordinary chemical batteries for some forms of work, but static machines are not used. For ward work accumulators connected with trollies are at present in use, but eventually a supply from the main may be laid on to the wards, if means can be devised of avoiding short-circuiting while using a supply from a fixed point with necessarily movable apparatus. In at least one recognised hospital much undesirable capital has been made out of the possession of some particular method or apparatus for applying electrically produced light, and to this are due some of the delusions of the public as to what benefit from electrical treatment at all can be looked for with any certainty. At the Middlesex Hospital this is not the case at all; the authorities have pinned their faith to no special form of instrument, but frankly recognise that the therapeutic uses of electricity are at present indeterminate. They have therefore provided themselves with specimens of all modern apparatus and are steadily trying them one against the other. In every case a careful record is kept of the precise circumstances under which treatment is undertaken and of its duration and apparent results. Furthermore, when the number of corresponding lesions in one patient makes it possible, the different effect of two forms of treatment simultaneously applied are carefully observed. This is the right spirit in which the matter should be undertaken. It offers a possibility of real progress, and a probability that before long order and real knowledge will be introduced into a line of treatment which is at present somewhat confused and largely empirical. It will certainly result in the clearing away of a large number of fallacies, and may possibly decide the question of whether the virtues of light depend upon some factor, or ray, common to all forms of it but present to a greater extent in some combinations than in others, or whether, on the other hand, therapeutic results are due to simple intensity of ordinary compound light or to a special combination of rays. The settlement of this point would be of the greatest benefit, as all the energy and money now expended over many fields of research could then be concentrated on the production either of the single factor in its least diluted form, or on the intensification and production of whatever compound light may be found to be most effective. The principal apparatus in use and under experiment at the Middlesex Hospital are two *x*-ray tubes with 12-inch coils, two of Sequiera's modification of the Finsen lamp, some discs containing bromide of radium, a Hewitt mercury-vapour lamp, two forms of high-frequency apparatus, and a Leslie Miller lamp. The *x*-ray apparatus are of Cox's latest model with mercury interruptors, and very powerful. Sequiera's modification of the Finsen lamp seems really a different apparatus altogether, being smaller, more convenient to handle, and economical in use. No means are used to shut off all but violet rays, and in essence it is an intensely brilliant arc-lamp, the light of which is applied to the part affected through a rock-crystal bull's-eye, all heat being abstracted by a shield containing running water.

The high-frequency machines are of two patterns. In the one the current from the main passes first into a motor, thence into a transformer, thence to a Rumkorf coil, and finally to two brass rods. One of the latter is movable, and the strength of the current to be diverted into the water cylinder by which it is to be applied to the person can be varied by increasing or diminishing the air resistance between their two extremities. In the other form (Cox's model) the current passes first into two Leyden jars, thence through a spark gap, and finally either through a solenoid alone or through both a solenoid and an Oudin's resonator. In the latter case the current is emitted from the bulb at the top in the form of a brush. If there is any advantage in this form over the older model it is that a current of greater rapidity and intensity can be used without undue shock. Hewitt's mercury-vapour lamp is a simple vacuum tube, about 18 inches long, containing a mercury reservoir. The latter is in communication with the negative wire from the galvanometer and the positive wire is twisted round the base. When the current is turned on the mercury is suddenly vaporised in the vacuo, and gives rise to a curious violet light entirely void of red. The therapeutic value of this light, like that given by the bromide of radium discs, is at present entirely unknown.

